# Immobilized uricase for automated assay of uric acid in serum

**DOI:** 10.1155/S1463924682000054

**Published:** 1982

**Authors:** Luis P. León, Joan B. Smith, Anne Yeung, Chien K. Yeh, Csaba Horváth

**Affiliations:** 1 Department of Clinical Chemistry Research and Development Technicon Corporation Tarrytown New York 10591 USA; 2 Department of Chemical Engineering Yale University New Haven Connecticut 06520 USA


Journal of Automatic Chemistry, Volume 4, Number 1 (January–March 1982), pages 11–16

### Immobilized uricase for automated assay of uric acid in serum

**Luis P. León, Joan B. Smith, Anne Yeung, Chien K. Yeh**

*Department of Clinical Chemistry Research and Development, Technicon Corporation, Tarrytown, New York 10591 USA,*

**and Csaba Horváth**

*Department of Chemical Engineering, Yale University, New Haven, Connecticut 06520, USA*

The wide use of bound enzyme coils with hexokinase and glucose-6-phosphate dehydrogenase in automated analysis for serum-glucose assay [1, 2] demonstrates the potential of open tubular heterogeneous enzyme reactors [3] in clinical analysis. Such reactors have also been prepared with other enzymes [4, 5] and in this paper the authors describe a continuous-flow method for uric acid assay with immobilized uricase, as well as some of the characteristics of bound uricase coils.

The classic alkaline phosphotungstate method for uric acid assay has been gradually replaced by enzymic methods that employ uricase and measure the concentration of one of the reaction products: allantoin or hydrogen peroxide [6–12]. The authors used tubes with immobilized uricase at the inner wall on Technicon's SMA and SMAC continuous-flow analysers and the reaction scheme is shown in figure 1. Dialysed uric acid enters the reactor coil and is oxidized in a reaction catalysed by

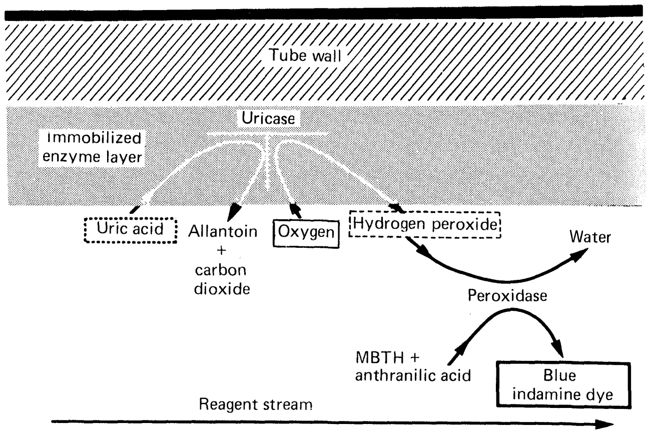

*Figure 1. Schematic illustration of ureate oxidation by uricase immobilized at the tube wall and the subsequent indicator reaction for hydrogen peroxide that takes place in the liquid phase.*

immobilized uricase. The concentration of hydrogen peroxide formed in the reaction is measured by the indicator reaction that involves the oxidative coupling of 3 methyl-2-benzothiazolinone hydrazone (MBTH) and anthranilic acid in the presence of peroxidase. The uric acid concentration is proportional to that of the blue indamine dye, which is measured colorimetrically at 570 nm.

### Materials and methods

### *Reagents and standards*

The *sample diluent* used was Technicon's T01-0992, which contains 1 g of ethylene-diaminetetraacetic acid (EDTA) disodium salt and 0·8 g EDTA ferric sodium salt (Technicon) dissolved in 1 l of phosphate buffer. The pH of the diluent was 7·3. The *borate buffer* was T01-0988, 0·5 mol/l at pH 9·2. For the *colour reagent* the contents of a vial of T01-1085 or T11-0989 colour reagent for uric acid (Technicon) was dissolved in 200 ml of water to make solutions for use on an SMAC and an SMA 12/60, respectively. Both reagents contain 3·06 g of anthranilic acid and 0·10 g MBTH/l. *Peroxidase reagent* was prepared by reconstituting a vial of lyophilized horse-radish peroxidase (T11-0579) with 200 ml of peroxidase reagent diluent (T21-0582) in order to obtain a solution with 16 kU of peroxidase activity/l.

To prepare the uric acid standard (1·0 g/l) 100 mg of uric acid (MBS reference reagent) and 100 mg of lithium carbonate was dissolved in 100 ml of distilled water. This stock solution is stable for about one month if refrigerated and protected from light. Working uric acid standards are prepared by diluting the stock solution to give 2, 4, 6, 8, 10, 12 and 15 mg of uric acid/dl.

The *bound uricase coil* was made by coating polyamide tubing with uricase from *Candida utilis*, according to the procedure described by Hórvath and Solomon [13]. The inner diameters and lengths of the bound enzyme tubes were 0·10 and 25 cm for the SMAC and 0·16 and 30 cm for the SMA. The tubes were coiled and mounted into 6 mm I.D. plastic sheath with proper connections to the appropriate SMA and SMAC cartridges.

*Serum samples:* pooled human serum was obtained from the Memorial Sloan-Kettering Medical Center (New York, N.Y. 10021) and stored at $-18°C$. The reference serum used was manufactured by Technicon.

### *Instrumentation and procedures*

Custom-built single-channel SMAC and SMA analysers were used for method development. A simplified flow-sheet, appropriate for both kinds of cartridges together with pertinent operational details, is shown in figure 2. Method performance characteristics were subsequently evaluated on the SMAC and SMA instruments generally used in clinical laboratories.

### *Evaluation of carry-over*

A set of nine samples, six of which had low uric acid concentrations and three high concentrations, was analysed in the order shown by the tracing in figure 5. The ratio of uric acid concentrations for the 'high' and 'low' samples was 10. Percentage sample interaction, C.O., was calculated by using the formula

$$C.O. = 100(A_7 - \bar{A}_L)/(\bar{A}_H - \bar{A}_L) \quad (1)$$

where $A_7$ is the absorbance at the flat of the tracing for sample No. 7, $\bar{A}_L$ is the average absorbance for sample Nos. 2, 3, 8 and 9 whereas $\bar{A}_H$ is the average reading for sample Nos. 5 and 6.

### Results and discussion

### *Evaluation of the method*

The studies were carried out with three different SMA 12/60 units and three different SMAC instruments, over 10 consecutive working days, in order to characterize the performance of

0142–0453/82/0401 0011 $05·00 © 1982 Taylor & Francis Ltd the new method under field conditions at three laboratories: Mt. Sinai Hospital (Baltimore, Maryland 21215); Lakeville Medical Labs (New York, N.Y. 10021); and Technicon's Reference and Evaluation Laboratory. In-house evaluation was based on a specially designed computerized protocol [14], which yields detailed information on precision, percentage recovery, accuracy, carry-over and drift on the analytical data obtained with SMA 12/60s and SMACs. Three samples were prepared with low, medium, and high uric acid concentrations by using human serum pool of low uric acid level as the diluent, and assayed. The results are listed in tables 1 and 2 and can be summarized as follows.

*Sensitivity*

The maximum sensitivity of this method with a fresh, 30 cm long immobilized-uricase coil was found to be 0·02 absorbance units/mg/dl uric acid under conditions employed on SMAC equipped with an 18 in. dialyser. A prolonged test for operational stability showed a slight decrease in coil activity after 20 days.

*Range of linearity*

Linear response was obtained with samples having uric acid concentrations up to 12 and 15 mg/dl for SMA and SMAC, respectively.

*Drift*

The average drift was found to correspond to a uric acid concentration of $\pm 0{\cdot}17$ mg/dl for both the SMA 12/60 and SMAC within the usual recalibration cycle of 10 and 47 specimens, respectively.

*Carry-over*

In a broad concentration range with the three samples, carry-over was found to be $4{\cdot}8 \pm 0{\cdot}57\%$ and $1{\cdot}0 \pm 0{\cdot}24\%$ for the SMAC and SMA 12/60, respectively.

*Correlation*

Over 100 serum samples, each approximately one week old, were randomly selected from a hospital population, and assayed in duplicate for uric acid in a random sequence at a rate of 10 samples per day over a period of 10 days. The present method with bound uricase coil was compared to both the manual uricase method with spectrophotometry at 293 nm and continuous-flow methods with phosphotungstate on Technicon analysers. Results of the statistical evaluation are in excellent agreement with those obtained by the other assay procedures as shown in table 3.

*Interferences*

Uric acid assay is generally subject to interference by certain components. For instance, in the automated method devised by Gochman and Schmitz [9], ascorbic acid seriously depressed colour development in the indicator reaction and similar observations were made by Klose *et al.* [12]. The authors used EDTA-iron complex [15] to counteract such interference. As seen in table 4, when the concentration of ascorbic acid is not higher than 5 mg/dl, interference is suppressed. Thus the use of the metal complex is effective in alleviating interference by ascorbic acid, the concentration of which is expected to be much lower than 5 mg/dl, and can minimize the effect of other reducing substances commonly found in serum.

Table 4 also shows the results obtained with a variety of other substances potentially present in serum. Human serum samples were spiked with the substances at concentrations corresponding to multiple-interference levels, and serum devoid of interfering substances was used as the reference. Allopurinol, bilirubin, creatinine, cysteine, glucose, hypoxantine, phenol, theophylline, triglycerides, heparin, salicylate, EDTA, oxalate and iodoacetate showed no clinically significant bias on the method at the upper limit of their expected serum concentration. On the other hand, clinically significant interference by the

*Table 1. Precision of uric acid assay with bound uricase coils on the SMA 12/60 and SMAC systems as evaluated from measurements with human serum pools having low, mid and high glucose concentrations.*

| Method | Level | Mean uric acid concentration (mg/dl) | Total precision (SD)[1] | Total precision (CV%) | Within-run precision (S.D.) | Within-run precision (CV%) |
|---|---|---|---|---|---|---|
| SMA | Low | 2·1 | 0·07 | 3·2 | 0·05 | 2·5 |
| | Mid | 7·7 | 0·09 | 1·2 | 0·09 | 1·2 |
| | High | 12·9 | 0·18 | 1·3 | 0·11 | 0·9 |
| SMAC | Low | 2·5 | 0·27 | 11·1 | 0·27 | 10·9 |
| | Mid | 9·4 | 0·27 | 2·8 | 0·27 | 2·8 |
| | High | 15·4 | 0·34 | 2·2 | 0·26 | 1·7 |

1. Includes day-to-day plus within-run precision data.

*Table 2. Accuracy of uric acid assay with bound uricase coils on the SMA 12/60 and SMAC systems as evaluated from measurements with human serum pools having low, mid and high glucose concentration. Theoretical uric acid values were obtained by measuring uric acid concentrations with the spectrophotometer at 293 nm.*

| Method | Level | Theoretical uric acid concentration (mg/dl) | Uric acid concentration recovered (mg/dl) | Deviation from expected uric acid concentration (mg/dl) | Uric acid recovered (%) |
|---|---|---|---|---|---|
| SMA | Low | 2·1 | 2·0 | −0·1 | 95·7 |
| | Mid | 7·4 | 7·7 | 0·3 | 104·6 |
| | High | 12·6 | 13·0 | 0·4 | 103·4 |
| SMAC | Low | 2·4 | 2·1 | −0·3 | 88·4 |
| | Mid | 9·1 | 9·4 | 0·3 | 104·4 |
| | High | 15·8 | 15·7 | −0·1 | 99·6 |

*Table 3. Correlation between the present method with bound uricase coils and other methods for uric acid assay in serum.*

| Continuous flow method with bound uricase coils *y* | Reference methods *x* | Number of assays | Regression equation | Correlation coefficient | Standard error of estimate |
|---|---|---|---|---|---|
| SMA 12/60 | Manual with uricase (spectrophotometer at 293 nm) | 105 | $y=0{\cdot}993x-0{\cdot}0$ | 0·997 | 0·2 |
| SMA 12/60 | SMA 12/60 with phosphotungstate | 291 | $y=1{\cdot}031x-0{\cdot}5$ | 0·991 | 0·3 |
| SMAC | SMA 12/60 with bound uricase coil | 342 | $y=0{\cdot}978x+0{\cdot}2$ | 0·999 | 0·21 |
| SMAC | SMA 12/60 with phosphotungstate | 343 | $y=0{\cdot}973x+0{\cdot}1$ | 0·999 | 0·21 |

substances listed in table 4 was found on both the SMA and SMAC.

### *Characterization of bound uricase coils*

It has been established that upon sequestering the enzyme the observed kinetics of reaction may be profoundly changed due to microenvironmental effects and diffusional resistances for the substrate and product molecules in the system. With open tubular heterogeneous enzyme reactors with continuous-flow analysers the peculiarities of diffusion and sample interaction in segmented flow have also to be considered [17]. The characteristics and operational behaviour observed with bound uricase coils are briefly described in the following.

### *Effect of substrate concentration*

Steady-state rates of uric acid were measured with bound uricase tubes of different lengths at various substrate concentrations. Using the SMA 12/60 instrument without the dialyser, uric acid was fed to the coil continuously in the reagent stream and the uric acid concentration was measured spectrophotometrically at 293 nm in both the feed and the effluent. Reaction rates were calculated from feed concentration, flow rate and conversion and normalized by the internal geometrical surface area of the coils. Lineweaver-Burk plots of the data are depicted in figure 3, which also shows plots of rate data obtained with uricase in free solution. In the latter case, the $K_m$ value was found to be $1{\cdot}4\times10^{-5}$ mol/l, in agreement with Pitts and Priest's data [18]. The difference observed between the kinetic behaviour of bound and free uricase is attributed to the relative slowness of radial mass transfer in enzyme coils under investigation [17]. Although the quasi-linear plots of the enzyme coil data in figure 3 cannot be used to extract meaningful kinetic parameters, they show the expected trend as far as the slopes and intercepts are concerned. When the tube length is increased from 3 to 10 cm the value of the reciprocal ordinate intercept, which some workers would consider the normalized 'apparent' $V_{max}$ of the reaction, decreases, while the 'apparent' $K_m$ value increases. These observations support the theory that predicts that the effect of diffusional resistances in the coil, which cause the observed kinetics to depart from the inherent kinetics of the enzymic reaction, become more pronounced with increasing tube length [3, 19].

### *Effect of coil length*

When the overall rate of reaction is determined by the rate of substrate diffusion to the enzymic wall, theory predicts that the

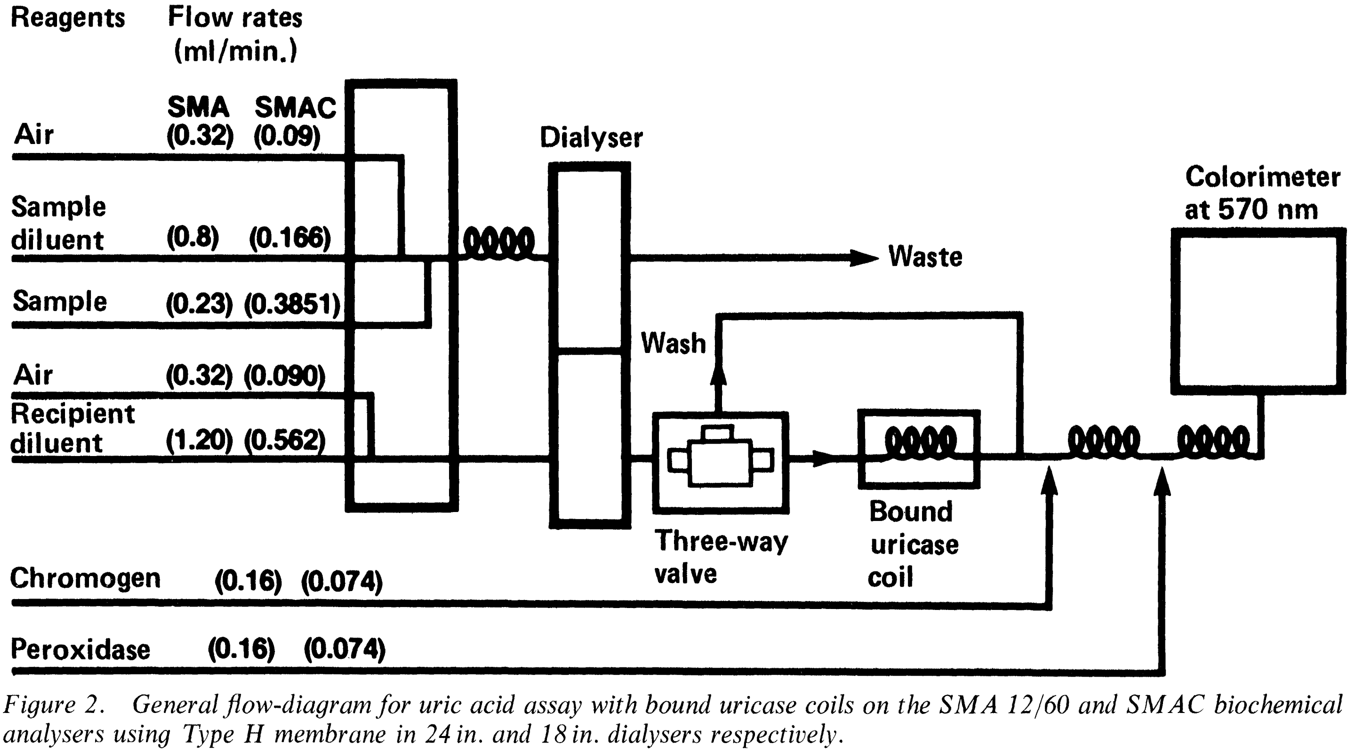

*Figure 2. General flow-diagram for uric acid assay with bound uricase coils on the SMA 12/60 and SMAC biochemical analysers using Type H membrane in 24 in. and 18 in. dialysers respectively.*

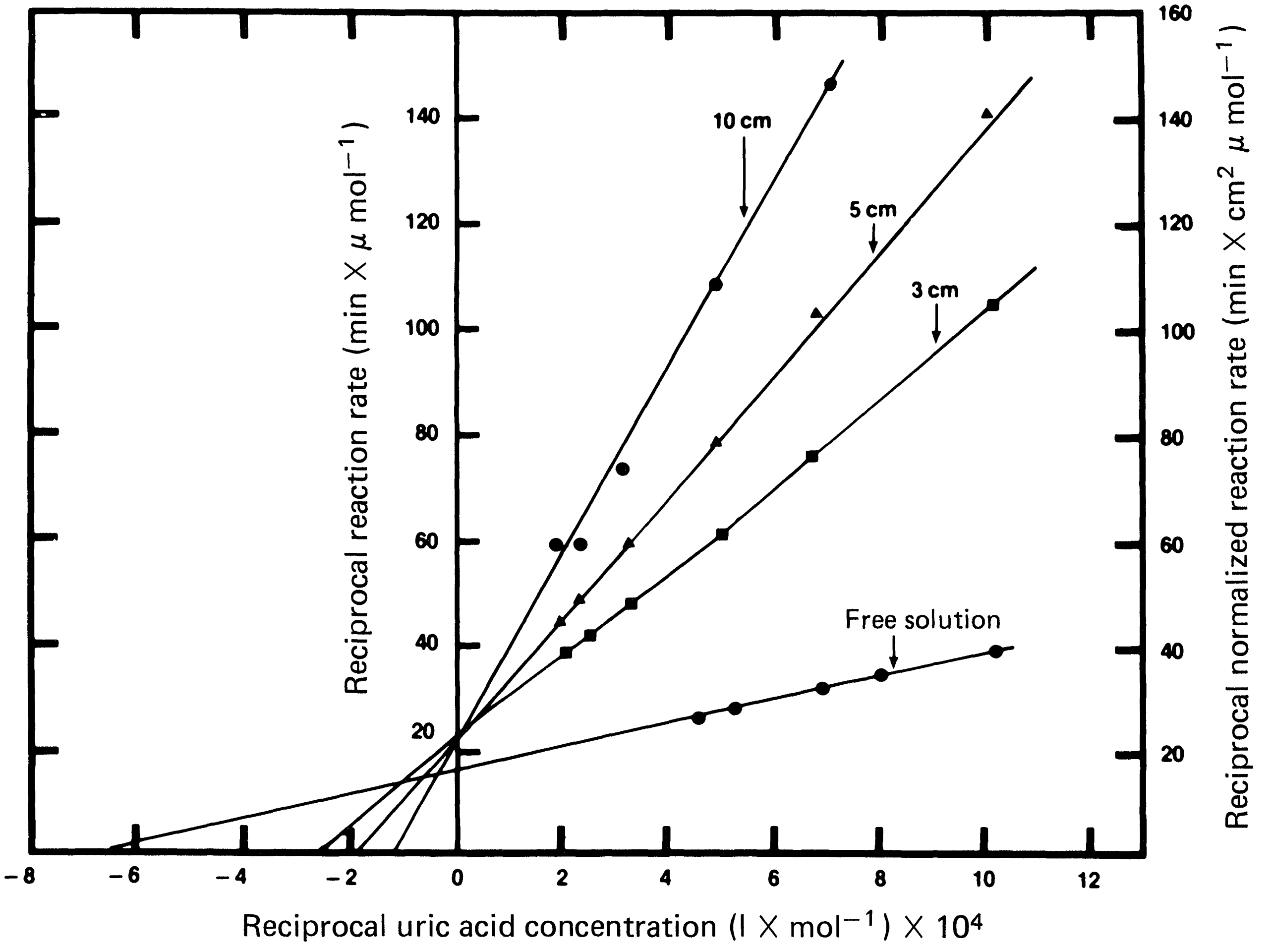

*Figure 3. Linewear-Burk plot of kinetic data obtained with uricase in free solution and with bound uricase coils of different lengths. Experiments were carried out on the same 12/60 by using continuous feed of uricase solution in 0·05 mol/l borate buffer, at pH 9·2, at a segmented flow rate of 1·2 ml/min. The concentration change for uric acid was followed spectrophotometrically at 293 nm. Human serum samples spiked with uric acid were used.*

normalized reaction rate is linearly dependent on $L^{-2/3}$, where $L$ is the tube length [13]. Results obtained with uric acid concentration in the range from 15 to 53 μmol/l under the conditions stated in figure 3 have indeed given straight lines on plots of the normalized reaction rate against $L^{-2/3}$. Since a linear relationship was observed with all tubes longer than 2 cm the authors conclude that with bound uricase coils of practical interest the overall reaction rate strongly affected by diffusional effects and is not determined by the inherent kinetics of the enzymic reaction.

The length dependence of conversion was also investigated under usual operating conditions of the SMAC, by using a human serum pool spiked with uric acid to obtain a substrate concentration of 15 mg/dl; the results are shown in figure 4. As seen, practically full conversion is obtained with SMAC coils longer than 60 cm. On the other hand, results of a similar study with the SMA showed a somewhat weaker dependence of conversion on the tube length; the conversion at any length was about 10% lower than that shown in figure 4. For the uric acid assay method on the SMAC and SMA 12/60 the length of bound uricase coils was selected as 25 and 30 cm, respectively. Such coils, which initially yield 90 and 80% conversion on the SMAC and SMA 12/60, have been found to represent the best compromise as far as sensitivity, sample interaction and the general quality of the tracing are concerned, as shown in figure 5.

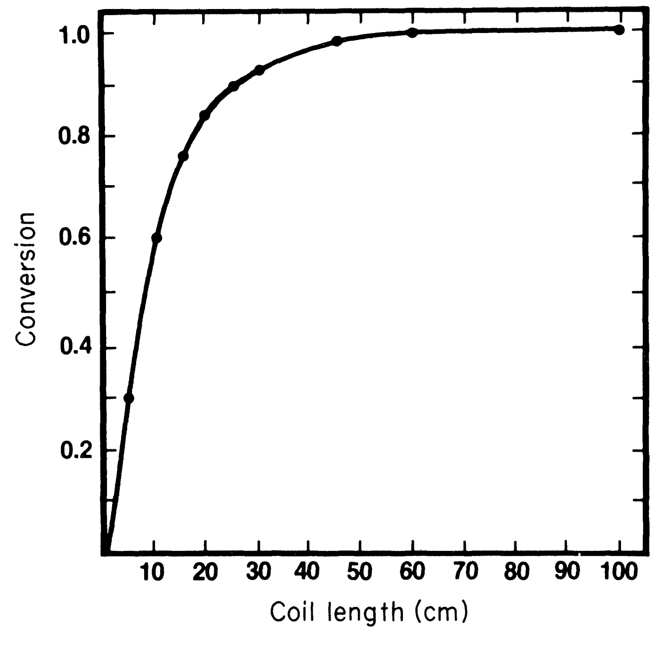

*Figure 4. Dependence of uric acid conversion on the length of bound uricase coils under conditions used on the SMAC.*

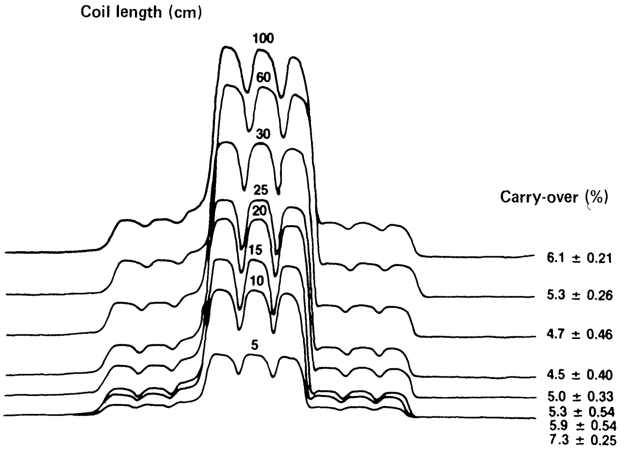

*Figure 5. Illustration of the effect of coil length on tracing quality obtained with bound uricase coil on a custom-built single-channel SMAC. The samples were from human serum pool and spiked with uric acid. Samples are numbered from left to right for the calculation of carry-over by using equation (1). Thus, sample Nos. 1, 2 and 3, low in uric acid are followed by Nos. 4, 5 and 6, which are high in uric acid, and by Nos. 7, 8 and 9 having low uric acid concentration.*

*Effect of flow rate*

As radial mass transfer in tubular enzymic wall reactors strongly increases with the velocity of segmented flow, the observed reaction rate in bound enzyme coils usually increases with flow rate. On the other hand, sample interaction is expected to be a complex function of the flow rate [17]. In practice, therefore, optimum flow rate is established empirically so that a compromise is found between maximum analytical sensitivity and minimum sample interaction. The effect of flow rate is illustrated by tracings in figure 6, which were obtained in uric acid assay by bound uricase coil on the SMAC. They show optimum results at 0·6 ml/min. Similar conditions led to the use of a flow rate of 1·3 ml/min. with SMA 12·60 cartridges.

*Effect of pH*

The pH activity profiles obtained with uricase in free solution and with a 25 cm long bound uricase coil for SMAC are depicted in figure 7. The data shown for the enzyme coil are average values obtained at uric acid concentrations in the range from 2·2 to 17·5 mg/dl. Figure 7 shows that the pH profile for the immobilized enzyme is much broader than that obtained with the enzyme in free solution. This observation can also be explained by the diffusion controlled nature of the reaction in the

*Table 4. Investigation of interference with uric acid assay by various substances which may be present in human serum. The data were obtained by using bound uricase coils on SMA and SMAC analysers (Technicon).*

| Interfering substance: Name | Interfering substance: Concentration (mg/dl) | Theoretical uric acid concentration (mg/dl) | Measured uric acid concentration (mg/dl) | Change in uric acid concentration (mg/dl) | (%) |
|---|---|---|---|---|---|
| Acetaminophen | 40 | 11·0 | 10·4 | −0·6 | 5·5 |
| Dopac | 2 | 9·8 | 9·0 | −0·8 | 8·2 |
| | 20 | 9·8 | 6·1 | −3·7 | 37·8 |
| L-Dopa | 2 | 10·9 | 10·5 | −0·4 | 3·7 |
| | 5 | 10·9 | 9·9 | −1·0 | 9·2 |
| | 20 | 11·0 | 8·3 | −2·7 | 24·5 |
| Gentisic acid | 2·5 | 11·2 | 10·9 | −0·3 | 2·7 |
| | 5 | 11·2 | 10·7 | −0·5 | 4·5 |
| | 10 | 11·2 | 10·4 | −0·8 | 7·1 |
| Hemoglobin (severe hemolysis) | | 5·2 | 4·6 | −0·6 | 11·5 |
| 6-Mercaptopurine | 25 | 9·8 | 9·1 | 0·7 | 7·1 |
| | 200 | 9·7 | 8·0 | −1·7 | 17·5 |
| α-Methyldopa | 10 | 9·8 | 8·9 | −0·9 | 9·2 |
| | 400 | 9·8 | 4·8 | −5·0 | 51·0 |
| Xanthine | 10 | 9·8 | 6·3 | −3·5 | 35·7 |
| | 300 | 9·8 | 1·0 | −8·8 | 89·8 |
| Sodium citrate | 1176 | 3·4 | 3·2 | −0·2 | 5·9 |
| Sodium fluoride | 1050 | 3·2 | 2·9 | −0·3 | 9·4 |
| Ascorbic acid | 5 | 4·8 | 5·0 | 0·2 | 1·4 |
| | 5 | 11·0 | 10·8 | −0·2 | 1·4 |
| | 10 | 4·8 | 5·2 | 0·4 | 8·3 |
| Acetaminophen | 40 | 12·0 | 11·7 | −0·3 | 2·5 |
| Dopac | 2·5 | 16·0 | 14·2 | −1·7 | 10·6 |
| | 20 | 16·0 | 9·0 | −7·0 | 42·7 |
| L-Dopa | 2·5 | 12·6 | 12·7 | 0·1 | 0·8 |
| | 5·0 | 12·6 | 12·7 | 0·1 | 0·8 |
| | 20 | 12·6 | 12·1 | −0·5 | 4·0 |
| Gentisic acid | 2·5 | 12·6 | 11·4 | −0·8 | 6·3 |
| | 5·0 | 12·6 | 10·2 | −2·4 | 14·0 |
| Hemoglobin | 500 | 11·3 | 10·8 | −0·5 | 4·4 |
| 6-Mercaptopurine | 25 | 16·1 | 15·9 | −0·2 | 1·2 |
| | 200 | 16·1 | 15·8 | −0·3 | 1·9 |
| α-Methyldopa | 25 | 16·0 | 15·7 | −0·3 | 1·9 |
| | 400 | 16·0 | 11·6 | −4·4 | 27·5 |
| Xanthine | 300 | 15·9 | 10·0 | −5·9 | 37·1 |
| Sodium citrate | 182 | 4·9 | 3·9 | −1·0 | 20·4 |
| Sodium fluoride | 1050 | 4·9 | 5·1 | 0·2 | 4·1 |
| Ascorbic acid | 3 | 11·8 | 11·6 | −0·2 | 1·7 |
| | 5 | 11·8 | 10·5 | −1·3 | 11·0 |
| | 10 | 11·8 | 7·9 | −3·9 | 33·1 |

bound uricase tube, i.e. the slowness of diffusion has a rate-equalizing effect on the overall reaction—in the pH range from 2·5 to 10·0 the observed rate changes very little. The linearity of the method has been found to depend strongly on the pH, however. Satisfactory analytical results changing the pH from this value in either direction resulted not only in decreasing linearity but also in a drop of sensitivity, and a deterioration of hydraulics. These observations mandate the use of the bound uricase coils at pH 9·2 in uric acid assay on both the SMAC and SMA 12/60.

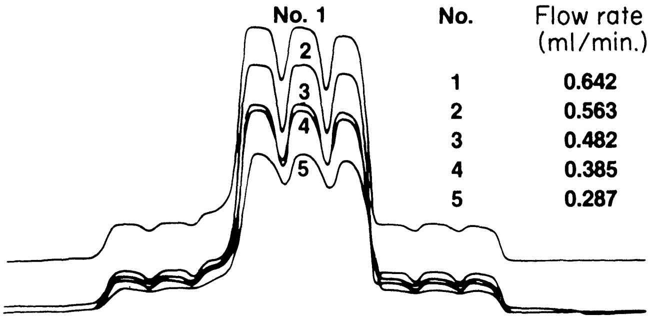

*Figure 6. Illustration of the effect of flow rate on tracing quality obtained with bound uricase coil on a custom-built single-channel SMAC. The samples were from human serum pool and spiked with uric acid.*

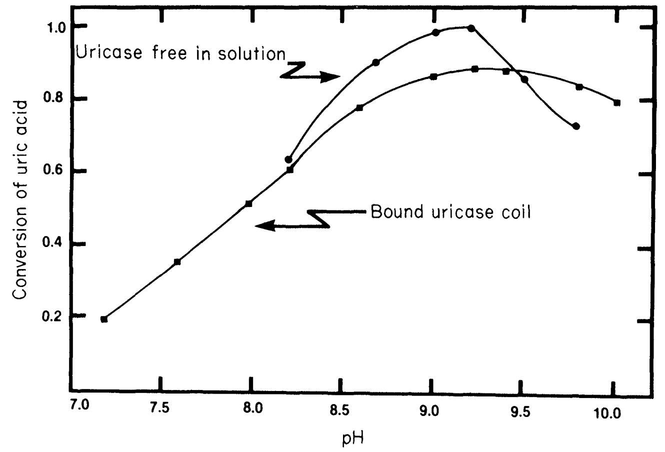

*Figure 7. pH profiles for bound and free uricase. Experiments were carried out on SMA 12/60 with samples having different uric acid concentrations. The data depicted here represent average values.*

### *Effect of temperature*

The rate of uric acid oxidation by bound uricase coils was practically unaffected by temperature in the range from 19 to 50°C at sample concentrations up to 7·3 mg/dl. At a sample concentration of 12·5 mg/dl the rate increase was less than 1% in the important temperature interval from 19 to 37°C. From the Arrhenius plot of these data we have calculated an activation energy of approximately 5 kcal/mol. This value is about half of that usually associated with enzymic reactions and also suggests that the overall rate of the reaction is strongly affected by diffusion, the activation energy of which is in the order of 2·5 kcal. The above findings suggest that under usual conditions the reaction can be conveniently carried out at room temperature and no thermostatting of the enzyme coil is necessary to obtain reproducible results.

### *Stability of bound uricase coils*

Enzyme coils from four different production lots were tested for operational stability on a two-channel SMA system during routine analytical work. The coils were not removed from the cartridges during the test period. Typical stability profile is illustrated in figure 8. The data show that after analysing 10 000 samples over a period of 35 days the initial activity of the coil declined by 24%. As the drop of analytical sensitivity was not accompanied by untoward changes in linearity and sample interaction the enzyme coils are considered to be sufficiently stable for use in continuous flow analysers.

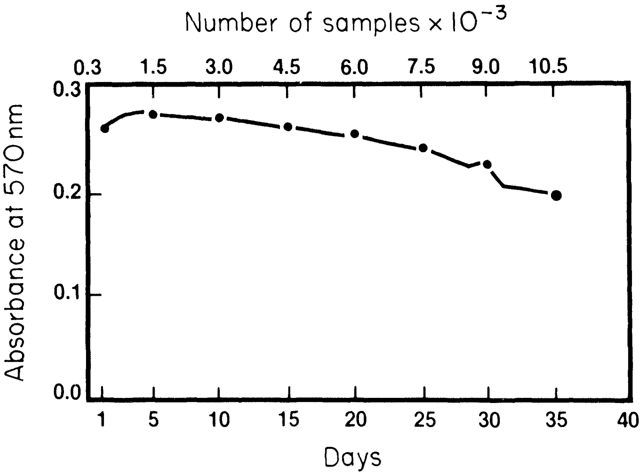

*Figure 8. Operational longevity of a typical bound uricase coil on the SMA 12/60 under usual conditions. The coils were not removed from the instrument during the time period shown.*

### References

1. Garber, C. C., Feldbruegge, D., Miller, R. C., Carey, R. and Neill, *Clinical Chemistry*, **24** (1978), 1186.
2. León, L. P., Chu, D. K., Snyder, L. R. and Horváth, Cs., *Clinical Chemistry*, **26** (1980), 123.
3. Horváth, Cs., Shendalman, L. H. and Light, R. T., *Chemical Engineering Science*, **28** (1973), 375.
4. Sundaram, P. V., Igloi, M. P., Wasserman, R. and Hinsch, W., *Clinical Chemistry*, **24** (1978), 1813.
5. Werner, M., Mohrbacher, R. J., Riendeau, C. J., Murador, E. and Cambiaghi, S., *Clinical Chemistry*, **25** (1979), 20.
6. Morgenstern, S., Flor, R. V., Kaufman, J. H. and Klein, B., *Clinical Chemistry*, **12** (1966), 748.
7. Steele, T. H., *Technical Bulletin of Registered Medical Technologists*, **39** (1969), 270.
8. Kageyama, N., *Clinica Chimica Acta*, **31** (1971), 421.
9. Gochman, N. and Smitz, J. M., *Clinical Chemistry*, **17** (1971), 1154.
10. Mietes, S., Thompson, C. and Roach, R. W., *Clinical Chemistry*, **20** (1974), 791.
11. Hunzicker, P. and Keller, H., *Journal of Clinical Chemistry and Clinical Biochemistry*, **13** (1975), 89.
12. Klose, S., Stoltz, M., Munz, E. and Portenhauser, R., *Clinical Chemistry*, **24** (1978), 250.
13. Horváth, Cs. and Solomon, B. A., *Biotechnology and Bioengineering*, **14** (1972), 885.
14. Daniel, C., *Journal of Quality Technology*, **7** (1975), 3.
15. Khan, M. M. T. and Martell, A. E., *Journal of the American Chemical Society*, **88** (1966), 3027.
16. Schrauzer, G. N. and Rhead, W. J., *International Journal of Vitamin and Nutritional Research*, **13** (1973).
17. Horváth, Cs. and Pedersen, H., *Advances in Automated Analysis; Technicon International Congress 1976*, Vol. I (Mediad, Tarrytown, New York, 1977), pp. 86.
18. Pitts, O. M. and Priest, D. G., *Archives Biochemistry and Biophysics*, **163** (1974), 359.
19. Horváth, Cs. and Engasser, J.-M., *Biotechnology and Bioengineering*, **16** (1974), 909.

